# Ab Initio Modeling of Mixed-Dimensional Heterostructures: A Path Forward

**DOI:** 10.1021/acs.jpclett.4c00803

**Published:** 2024-05-10

**Authors:** Jannis Krumland, Caterina Cocchi

**Affiliations:** † Institute of Physics, Carl von Ossietzky Universität Oldenburg, 26129 Oldenburg, Germany; ‡ Physics Department and IRIS Adlershof, Humboldt-Universität zu Berlin, 12489 Berlin, Germany

## Abstract

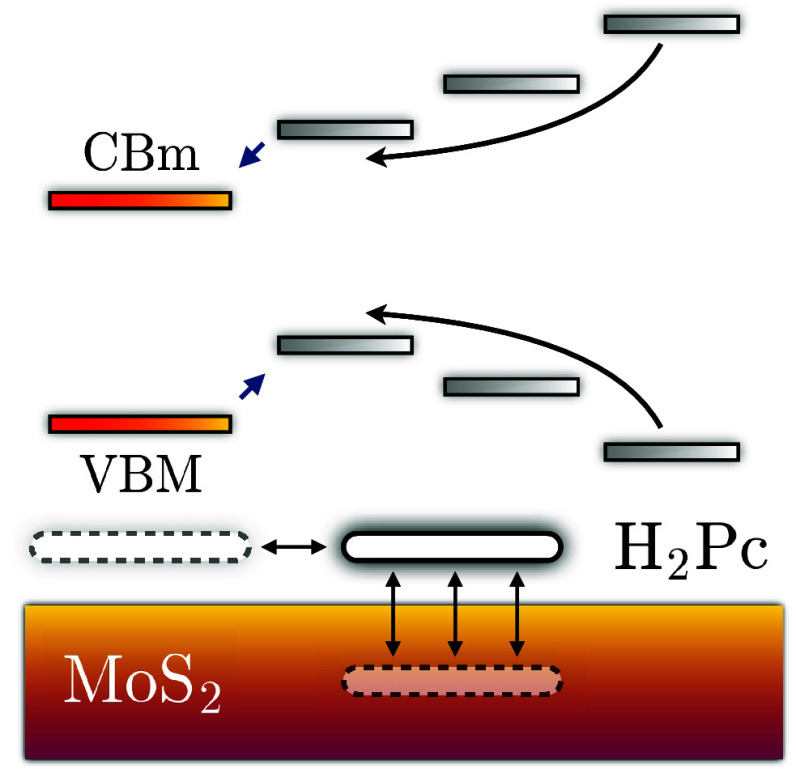

Understanding the electronic structure of mixed-dimensional heterostructures is essential for maximizing their application potential. However, accurately modeling such interfaces is challenging due to the complex interplay between the subsystems. We employ a computational framework integrating first-principles methods, including *GW*, density functional theory (DFT), and the polarizable continuum model, to elucidate the electronic structure of mixed-dimensional heterojunctions formed by free-base phthalocyanines and monolayer molybdenum disulfide. We assess the impact of dielectric screening across various scenarios, from isolated molecules to organic films on a substrate-supported monolayer. Our findings show that while polarization effects cause significant renormalization of molecular energy levels, band energies and alignments in the most relevant setup can be accurately predicted through DFT simulations of the individual subsystems. Additionally, we analyze orbital hybridization, revealing potential pathways for interfacial charge transfer. This study offers new insights into hybrid inorganic/organic interfaces and provides a practical computational protocol suitable for scaled-up studies.

In recent years, hybrid systems constituted by two-dimensional transition-metal dichalcogenides (TMDCs) and organic adsorbates have been extensively explored, attempting to exploit and combine the individual strengths of atomically thin semiconductors and carbon-conjugated molecules. In such mixed-dimensional heterojunctions, bright excitons with hybrid character have been observed,^1−4^ giving rise to an charge-separated state immediately upon photoabsorption. More commonly, dynamical charge transfer (CT) takes place in such systems, where the exciton is formed on either side of the interface and subsequently separated by electron or hole migration to the other subsystem.^2,5−11^ At other interfaces, the entire exciton is transported across the interface, representing a transfer of energy.^12−14^ Both charge and energy transfer in hybrid heterostructures have been exploited in various prototypical devices.^11,15−17^

The leverage of the full potential of such interfaces requires a detailed understanding of their electronic structure. At this point, atomistic modeling comes into play. First-principles simulations without empirical parameters provide an independent point of comparison to experiments and have been extensively used for both predictions and rationalization of measurements.^18−28^ However, mixed-dimensional interfaces still represent a challenge for density functional theory (DFT), which is the most popular quantum-mechanical *ab initio* method to deal with such systems thanks to its ability to handle hundreds to thousands of atoms. The bottleneck lies in the standard approximations of DFT, which are computationally efficient but do not capture an essential part of the interaction between substrates and adsorbates, namely the emergence of image potentials due to polarization of the other subsystem.^29^ Image potentials have a substantial impact on the energy levels of adsorbed molecules^30−34^ and therefore cannot be neglected. They are captured by the *GW* approximation from many-body perturbation theory,^35,36^ which consequently has become the state-of-the-art approach for the calculation of band gaps and level alignments at such interfaces.^37^ However, (non-self-consistent) *GW* simulations struggle with hybridized orbitals,^38^ are not trivial to conduct, and are computationally expensive, often requiring cutting corners.^39^ This is particularly true if one tries to account for the presence of substrates that are part of most experimental setups. In times when automatization, high-throughput simulations, and artificial intelligence take hold, more pragmatic and robust computational alternatives are desirable.

One way of mitigating the computational complexity of many-body simulations of inorganic/organic interfaces is the adoption of electrostatic models. Such models are able to successfully correct for long-range polarization, as long as a corresponding analytical expression for the image potential is available.^30,40−42^ This encompasses the scenarios of isolated molecules on the surface of a bulk material and isolated slabs.^25,43^ In previous work, we established a framework to include such corrections directly in DFT calculations through an expansion of the polarizable continuum model (PCM),^44^ named LayerPCM,^45^ which extends the range of the environments that can be modeled with the PCM to anisotropic stratified substrates, automatically accounting for the inherently nonlocal screening within such setups.^46^ By determining the frontier energy levels as total-energy differences through the so-called Δ*S*CF scheme, both the image potential contribution to the self-energy and orbital relaxation are captured simultaneously and self-consistently, going beyond the already quite successful schemes based on frozen-orbital model corrections. These Δ*S*CF+PCM calculations predict molecular frontier energies with an accuracy similar to *GW*,^38,47^ which is unsurprising given that the captured physical mechanisms are similar and Δ*S*CF is generally known to be competitive with *GW* for nonperiodic systems.^48,49^

In this work, we adopt a mixed first-principles methodology based on DFT as well as *GW*, Δ*S*CF, and the PCM to calculate the electronic structure of mixed-dimensional heterostructures formed by free-base pthalocyanine molecules (H_2_Pc) adsorbed on monolayer molybdenum disulfide (MoS_2_). We discuss the important role of dielectric screening occurring within molecular films, at interfaces, and due to substrates. The renormalization of molecular energy levels due to image charges in the substrate is found to be relevant mainly in the limit of low molecular coverage, while the energy levels of a deposited molecular film or crystal are not strongly affected. Based on these findings, we propose a robust and computationally cheap protocol for reproducing the electronic structure through the combination of subsystem results obtained with the widespread PBE, HSE, and PBE0 exchange-correlation functionals. Comparison to literature reports suggests this method to be generally transferable to other hybrid inorganic/organic interfaces. Going beyond the frontier energy level alignment, we showcase band unfolding techniques that allow us to pinpoint hybridized states, which may turn out to play a key role in interfacial charge-transfer processes.

Before delving into the analysis of hybrid interfaces, we first consider the isolated subsystems, starting with the H_2_Pc molecule. We employ the Δ*S*CF method, computing the ionization (IP) potentials as *E*_*N*–1_ – *E*_*N*_, where *E*_*N*–1_ and *E*_*N*_ are the ground-state energies of the single cation and the neutral molecule, respectively, and electron affinities as *E*_*N*_ – *E*_*N*+1_, where *E*_*N*+1_ is the ground-state energy of the molecule with an additional electron. Details of our simulations are provided in the Supporting Information (SI). For molecules in the vapor phase, modeled as isolated entities *in vacuo*, ΔSCF calculations with the semilocal PBE functional^50^ (ΔPBE) predict an IP of 6.41 eV and an EA of 2.17 eV, corresponding to a fundamental gap of 4.24 eV [Figure 1a]. The IP is in quantitative agreement with results from photoelectron (PE) spectroscopy for H_2_Pc (6.41 eV^51^), as well as for copper (CuPc) and zinc phthalocyanine (ZnPc), which have similar π frontier orbitals (6.34–6.38 eV^51−53^). To the best of our knowledge, measured results of EA are not yet available.

**Figure 1:**
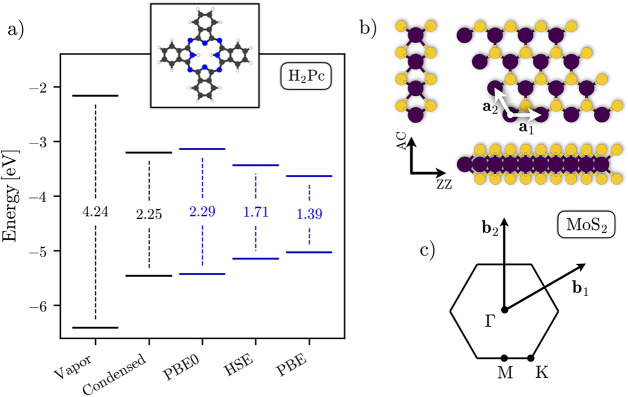
(a) Frontier energy levels and fundamental gap of the H_2_Pc molecule (geometry in the inset panel, with H, C, and N atoms in white, gray, and blue, respectively), calculated with ΔPBE (vapor phase), ΔPBE+PCM (condensed phase), and three PBE-based exchange-correlation functionals (PBE0, HSE, and PBE functionals without PCM). (b) Top and side views of monolayer MoS_2_, with armchair (AC) and zigzag (ZZ) directions as well with the primitive lattice vectors **a**_1_ and **a**_2_ highlighted. Mo and S atoms are depicted in violet and yellow, respectively. (c) Brillouin zone associated with the hexagonal lattice of MoS_2_, including the primitive reciprocal lattice vectors **b**_1_ and **b**_2_ and the high-symmetry points Γ, M, and K.

To quantify the effect of screening on the energy levels of organic films or crystals,^54,55^ we first determine the dielectric constant ε of condensed-phase H_2_Pc with a Clausius–Mossotti-like equation.^56,57^ This relates the microscopic polarizability of a single H_2_Pc molecule, calculated with DFT, to the macroscopic dielectric constant, yielding ε = 5.32 (details in the SI). This value is used to parametrize the PCM potential for ΔPBE+PCM calculations, using the standard implementation of PCM that assumes the molecule to be surrounded by an extended isotropic medium. A polarization term is added to the total energy, accounting for the electrostatic interaction between the molecule and the polarization charges forming around it. This causes a reduction of the IP from 6.41 to 5.46 eV with respect to vapor phase, as well as an increase of the EA from 2.17 to 3.21 eV, symmetrically narrowing the fundamental gap from 4.24 to 2.25 eV [Figure 1a]. It is worth noting that PE measurements of H_2_Pc, CuPc, and ZnPc films, which all have comparable π-dominated frontier energy levels, suggest somewhat lower values for the IP (4.82–5.20 eV with an average of 5.07 eV^7,58−64^). This discrepancy can be ascribed to two experimental factors. The first is the common practice of defining the IP as the very onset of the PE spectrum,^7,61−65^ which differs significantly from the maximum of the first peak, which our value corresponds to. The comparatively large difference between the onset and the maximum reflects increased peak broadening in the condensed phase, which depends on a myriad of parameters, as manifest in the broad range of reported experimental values in contrast to the narrow distribution of results for the vapor-phase IPs. The second factor is the dependence of the energy levels on the exposed surface. Molecular films or crystals with a lateral (edge-on) surface have a different vacuum level than those with a cofacial one,^66^ which reflects the fact that the work function is a surface-sensitive quantity.^56^ As our condensed-phase simulations are based on single molecules, they implicitly assume a film surface that corresponds to the prevalent surface of single molecules. As single H_2_Pc molecules have significantly more cofacial than lateral surface area, we therefore model a film with a cofacial surface. To estimate theoretically the effect of noncofacial surface orientations, the electrostatic potential around stacks of atomistically modeled molecules should be considered.^66^ Experimentally, it was found that the energy levels of CuPc adsorbed edge-on are shifted up by 0.40 eV or down by 0.81 eV compared to face-on adsorbed molecules, depending on whether the molecule is terminated by H or F.^64^ Similar shifts can be expected for H_2_Pc.

Summarizing, we thus have in condensed phase (i) a polarization-induced reduction of the fundamental gap of the molecule, (ii) a PE peak broadening due to other intermolecular interactions and disorder, and (iii) a rigid shift of the energy scale due to the surface-dependent work function.

Next, we contrast the ΔSCF results with the KS electronic structure obtained with three popular PBE-based exchange-correlation functionals: PBE itself, the global hybrid functional PBE0^67^ with a 25% exact exchange admixture, and the range-separated hybrid functional HSE,^68,69^ in which the amount of exact exchange decreases from 25% at short-range to 0% at long-range. We do not include PCM in these calculations, considering just the isolated molecules, as the inclusion of PCM does not significantly affect the KS orbital energies of neutral molecules.

None of the three functionals above provides KS frontier energies that are even remotely close to the vapor-phase IP and EA, with the predicted energy gaps being significantly smaller than the experimental values [Figure 1a]. However, the energy levels from PBE0 are remarkably close to those of the molecules in the condensed phase, suggesting that PBE0 results can generally be interpreted as estimates thereof. A corresponding observation has been made for the similar B3LYP functional,^70^ which reproduces quite well frontier-orbital energies of organic solids from (inverse) PE spectroscopy across a wide range of molecules.^71^ This finding can be rationalized in terms of screening: it has been shown that hybrid functionals predict accurately the energies of organic solids if the long-range exact-exchange fraction is adjusted to 1/ε.^72^ According to this logic, B3LYP and PBE0 with their flat 20–25% of exact exchange correspond to ε = 4 to 5, which fits organic solids of medium-sized molecules quite well. In conventional inorganic semiconductors, on the other hand, ε is significantly higher such that 1/ε → 0, explaining why HSE with its diminishing long-range exact exchange performs well for such systems.^73−75^

We continue with the analysis of the second subsystem, the MoS_2_ monolayer [Figure 1b-c]. For the moment, we focus exclusively on the band edges, i.e., the energies of the valence band maximum (VBM) and the conduction band minimum (CBm). For extended systems, ΔSCF is not an option and we rely instead on the *GW* approximation.^35^ More specifically, we calculate a single-shot *G*_0_*W*_0_ correction to the PBE energy levels, using a full-frequency integration (details in the SI). The PBE starting point has the VBM and CBm at −5.89 eV and −4.24 eV, respectively, corresponding to a band gap of 1.65 eV [Figure 2a]. The *G*_0_*W*_0_ correction brings these values to −6.34 eV (VBM) and −3.67 eV (CBm), opening up the band gap to 2.67 eV, in good agreement with other many-body simulations.^76^ Interestingly, the KS gap of MoS_2_ as calculated with the PBE0 functional is quite similar to the *GW* quasiparticle gap [Figure 2a]. However, the absolute energies of the orbitals are too low, as generally observed for hybrid functionals.^38^ Since absolute energies are of crucial importance in the study of interfaces, the inability of hybrid functionals to predict them represents a major drawback, which is another reason that results from fully atomistic DFT simulations of hybrid interfaces have to be analyzed with caution. In fact, taking the *GW* result as a benchmark, PBE reproduces the midgap position more accurately than PBE0, confirming previous reports of semilocal functionals predicting the center of the gap quite well.^77−79^ Thus, as a pragmatic approach for obtaining band structures with reasonably accurate absolute energies, we can take the PBE0 result and center it around the midgap position from PBE.

**Figure 2:**
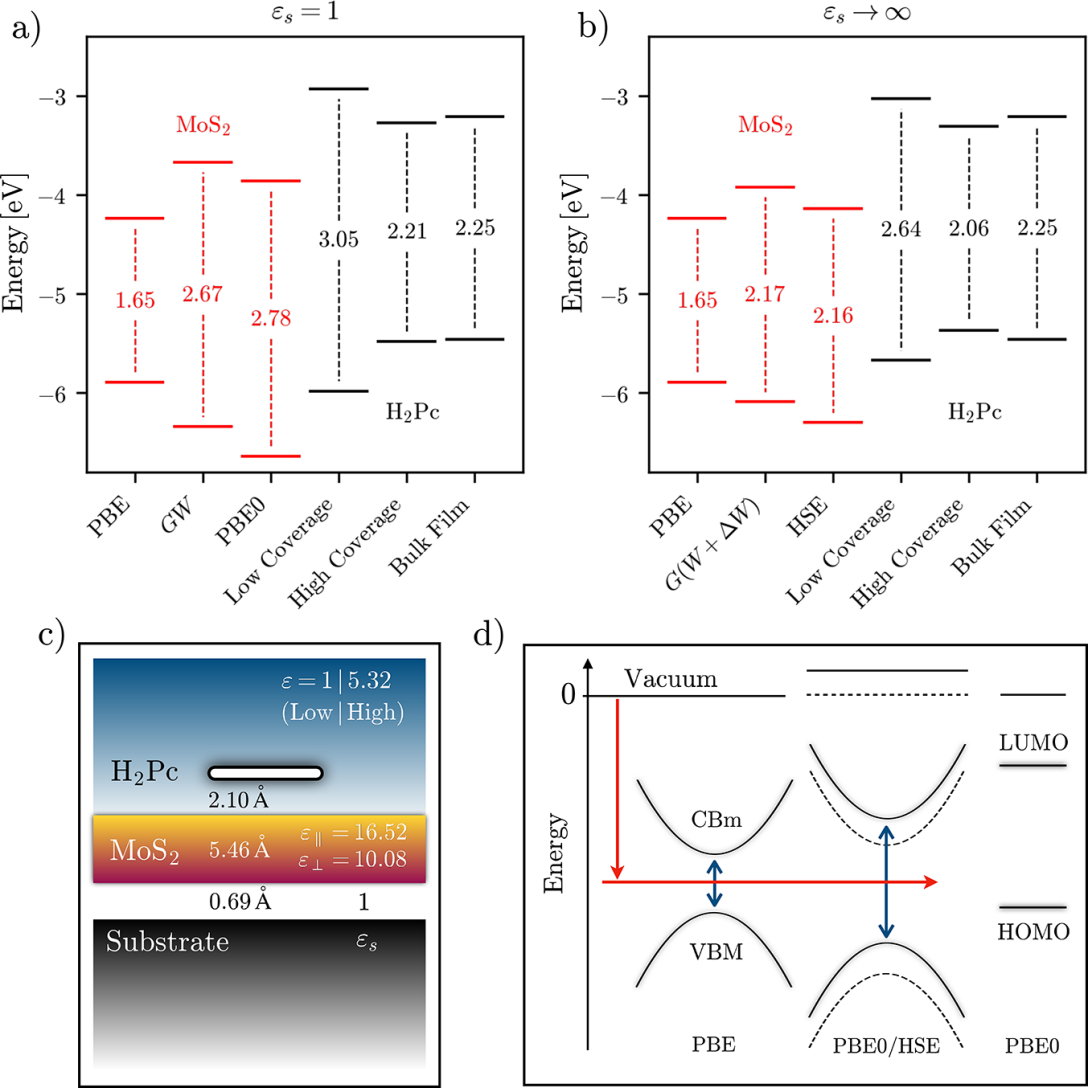
Energy level alignment between monolayer MoS_2_ and H_2_Pc, assuming the two limiting cases (a) ε_*s*_ = 1 and (b) ε_*s*_ → ∞ for the dielectric constant of an underlying substrate. For MoS_2_, the Kohn–Sham band edges obtained with the PBE and PBE0 (a) or HSE (b) functionals are shown and compared to full-frequency *G*_0_W_0_@PBE results, using a *GΔW* self-energy correction for ε → ∞. For the molecules, isolated adsorbed molecules (low coverage) and adsorbed films (high coverage) are accounted for and compared to the values for bulk molecular films. The corresponding model setups are shown in (c), including the parameters used for the construction of the LayerPCM response function. (d) Pictorial representation of the PBE/HSE/PBE0 simulation protocol.

When a substrate is added to the picture, energy levels change substantially. The band gap of MoS_2_ is reduced by an amount that can be estimated with a static polarization model since the long-range polarization is mainly determined by the static limit of the dielectric function ε_*s*_ of the substrate.^80^ This model, referred to as *GΔW* and described in the SI (Figure S3) as well as in ref (38), is in large parts physically and mathematically equivalent to the LayerPCM approach that we adopt below for adsorbed molecules. It does not account for the detailed spatial distribution of the Bloch functions, which, however, has been shown to be of little importance in this context.^81^ In the limiting case of highly polarizable substrates (ε_*s*_ → ∞), the gap is reduced from 2.67 to 2.16 eV^38^ [Figure 2b]. In spite of indications about the importance of vertex corrections for the determination of absolute band energies,^82,83^ we find the VBM energy of −6.09 eV from substrate-corrected *GW* to agree well with the experimental value of −6.10 eV, which we obtained by averaging results of several PE measurements on substrate-adsorbed MoS_2_ reported in the literature^6,13,15,20,84−89^ (values in the SI). The band gap of substrate-sustained MoS_2_ turns out to be very similar to the band gap given by the HSE functional, although, again, absolute energies from the hybrid functional are too low [Figure 2b]. As before, a better estimate is obtained by centering the HSE bands around the midgap position from PBE.

We now move on to the hybrid heterostructures. The simplest scenario is given by a single H_2_Pc molecule adsorbed on an isolated MoS_2_ monolayer [Figure 2c with ε = ε_*s*_ = 1]. As in the organic-film simulation, we estimate the molecular frontier energy levels with ΔPBE+PCM, adopting the LayerPCM response function for the latter. This leads to a significant reduction of H_2_Pc’s fundamental gap, from 4.24 eV in the vapor phase to 3.05 eV on MoS_2_ [Figure 2a], as a consequence of the well-known image-charge effect.^30^ A converse local band gap narrowing of MoS_2_ due to additional screening from the adsorbed molecule is negligible. We can thus put the energies of the highest occupied (−5.98 eV) and lowest unoccupied molecular orbitals (−2.93 eV) into relation with the pristine MoS_2_ band edges (VBM = −6.34 eV, CBm = −3.55 eV), revealing a type-II level alignment [Figure 2a]. These energies are in good agreement with values obtained from atomistic *G*_0_*W*_0_@PBE simulations of the entire hybrid system.^90^

In experimental practice, the scenario of isolated adsorbed molecules, i.e., very low coverage, is relatively rare and often does not even produce measurable optical signals.^8^ Typically, several organic layers are deposited,^5,6,8−10,15−17,91^ forming what is more accurately described as hybrid inorganic/organic interfaces rather than adsorbed molecules. We consider an H_2_Pc molecule directly adjacent to a MoS_2_ layer but surrounded by other molecules, modeled as a dielectric continuum. We refer to this setup as “high coverage” [Figure 2c with ε = 5.32]. The high-coverage scenario gives rise to frontier energy levels that are almost identical to the bulk film case [Figure 2a]. While the MoS_2_ layer has higher dielectric constants than the film, the lack of an underlying substrate (ε_*s*_ = 1) compensates the corresponding increase in screening. Although shielded by the MoS_2_ monolayer, single adsorbed H_2_Pc molecules are noticeably affected by a substrate with ε_*s*_ → ∞ [Figure 2b], further reducing the fundamental gap to 2.64 eV, from 3.05 eV for ε_*s*_ = 1. For films, this decrease is less pronounced, from 2.21 to 2.06 eV. We note that these values are close to the experimental result of 2.10 eV for an H_2_Pc film deposited on MoS_2_.^15^

We therefore find that the molecular energy level renormalization through polarization of MoS_2_ and its substrate is mostly relevant for low coverage, down to isolated molecules. Energy levels of deposited organic films, on the other hand, are not substantially affected, with a small change (∼0.1 eV) directly at the interface. The rather insensitive condensed-phase energy levels are predicted reasonably well with PBE0 simulations of single molecules *in vacuo*. Meanwhile, absolute values of band energies in MoS_2_ can be calculated with PBE0 (free-standing) or HSE (substrate-sustained), using a PBE result to establish the midgap position. Thus, a few inexpensive simulations of the individual subsystems can provide a good first approximation for the interfacial energy level alignment of the relevant high-coverage case [Figure 2d].

From the level alignment alone, different interfacial properties can be deduced. The amount of ground-state CT is related to the energy gap between the highest occupied state of one subsystem and the lowest unoccupied state of the other: the smaller the gap, the larger the amount of charge transfer. In the considered H_2_Pc@MoS_2_ system, the gaps are >1 eV in both directions, suggesting negligible CT, in agreement with experiments.^15,92^ We note that in the case of much smaller offsets, the resulting ground-state CT would lead to the formation of a dipole layer. Such layers give rise to different vacuum energies for both subsystems and therefore affect the level alignment with the levels of the electron-donating (-accepting) subsystem shifted down (up) in energy. As we neglect interactions between the subsystems beyond the polarization contribution, this effect is not explicitly accounted for in our simulations and we are limited to qualitative predictions in this regard. However, for physisorbed organic molecules without particularly strong donating or accepting capabilities, corresponding shifts are typically small (∼0.1 eV).^23^

While the energy level alignment is arguably the most important characteristic of an interface, there is a mounting number of observations that require the consideration of other aspects, including nonfrontier states and orbital hybridization.^93^ To expand our analysis in this direction, we introduce the related concepts of band structure unfolding and wave packet representation. Since the molecular potential does not have the translational symmetry of the hexagonal lattice of MoS_2_, the orbitals φ of H_2_Pc do not have definite **k** vectors. However, they can be constructed as a wave packet by superposing multiple Bloch states of MoS_2_ with different wave vectors **k**:
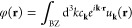
1where *c*_**k**_ is the superposition parameter associated with the Bloch vector **k** within the Brillouin zone (BZ) of MoS_2_, and *u*_**k**_ is a function with the translational symmetry of the MoS_2_ lattice. The spectral weights |*c*_**k**_|^2^ can be calculated with a projection technique, which does not require explicit knowledge of *u*_**k**_.^94^ Here, we symmetrize |*c*_**k**_|^2^ by averaging it over azimuthal molecular orientations starting at 15° in steps of 30°, giving it hexagonal symmetry [see Figure S2a]. This is appropriate for ensembles of molecules with randomized orientations, though it often may be worthwhile to analyze the orientation dependence in more detail. Due to complicated folding processes, the same orbital may turn out to have strongly direction-dependent wave packet representations [Figure S2a].

Plotting |*c*_**k**_|^2^ for all energies along a high-symmetry path in the BZ of MoS_2_, we obtain the equivalent of an unfolded band structure,^95^ which, if computed for atomistically modeled interfaces, contains rich information about intersubsystem orbital hybridization. However, it has been shown that it is not necessary to simulate the entire interface explicitly to predict the occurrence of orbital hybridization, which is predicated on just a few properties of the subsystem wave functions.^24,96^

To exemplify, we put the |*c*_**k**_|^2^ for H_2_Pc in relation to the band structure of MoS_2_ [Figure 3a]. Most of the occupied π orbitals are found to be represented by Bloch states close to K and M; the spectral weight of the HOMO of H_2_Pc is mainly concentrated at the K point and negligibly small at the zone center. This is most clearly seen upon plotting |*c*_**k**_|^2^ over the entire Brillouin zone [Figure 3b, left panels], producing an image similar to angle-resolved orbital PE spectra,^97^ though with high-momentum components folded into the BZ [Figure S2a]. The folding effectively turns momentum maps into crystal momentum maps. Experimental results for CuPc^98^ and FePc^99^ π orbitals show an absence of low-momentum contributions (|**k**| ≤ 1 Å^–1^). Thus, non-negligible low-momentum features in the interior of the BZ in Figure 3b are the result of folding. When tilting the molecules out of the face-on position, the HOMO starts producing a signal also at Γ [Figure S2b]. The predominant localization at K implies the possibility of a direct optical transition between the HOMO of H_2_Pc and the CBm of MoS_2_ with an in-plane transition dipole moment [Figure 3a, left arrow], giving rise to a CT excitation. Indeed, it is possible that the below-onset absorption band found for CuPc^1^ and ZnPc^2^ can be traced back to HOMO → CBm transitions. However, such CT excitations tend to have a very small oscillator strength as a result of negligible overlap between the spatially segregated electron and hole states. Instead, it has been suggested that bright charge-transfer excitations in heterojunctions can only exist in the presence of orbital hybridization, which leads to delocalized states that can exhibit substantial overlap with states of either subsystem.^100^ This leads us to the analysis of the lowest unoccupied molecular orbital (LUMO).

**Figure 3:**
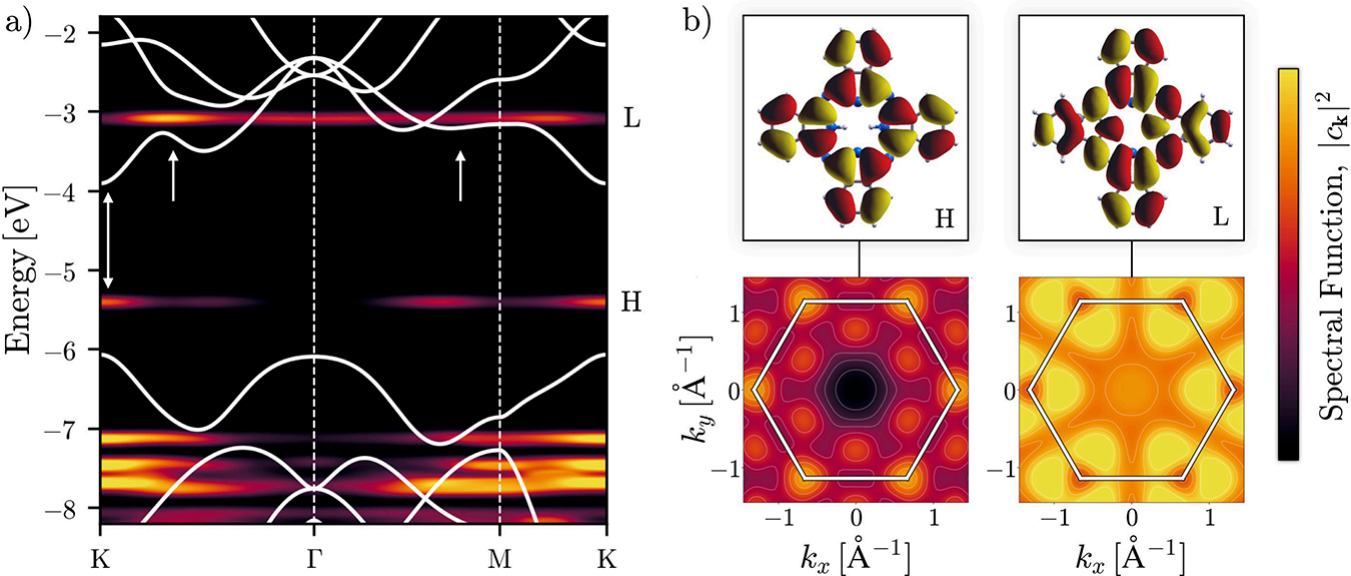
(a) Energy levels of H_2_Pc (calculated with PBE0) mapped into the Brillouin zone of MoS_2_ and plotted along the high-symmetry path, overlaid by the band structure of MoS_2_ (obtained by centering an HSE band structure around the midgap position from PBE). The arrows highlight points of interest, addressed in the main text. (b) Frontier orbitals of H_2_Pc in real space (top) and mapped into the Brillouin zone (bottom).

The spectral function associated with the effectively doubly degenerate LUMO of H_2_Pc^101^ is distributed more uniformly throughout the BZ, with some accumulation close to K [Figure 3a, center arrow, and Figure 3b]. The MoS_2_ states around this maximum are prone to interactions with adsorbates due to their increased atomic S character compared to the almost exclusively Mo-based CBm (meaning increased localization at the surface of MoS_2_^24^), which also manifests in strong band splitting in multilayer TMDCs.^102^ While some interactions between the LUMO and the MoS_2_ may occur at this point, strong mixing is prevented by their relatively large energetic separation. However, the electronic states of H_2_Pc and MoS_2_ overlap within an extended region between Γ and M [Figure 3a, right arrow]. Here, the degenerate molecular orbital interacts with multiple states of MoS_2_, giving rise to a manifold of hybrid states, each predominantly localized on MoS_2_, but with a small chance of electron scattering toward the H_2_Pc. Following the line of reasoning of ref (100), the bright hybrid excitations reported in refs (1) and (2) possibly correspond to a transition from the HOMO to such LUMO/MoS_2_ hybrid states. This demonstrates the possibility to deduce interface properties well beyond the energy level alignment from just subsystem calculations.

In summary, we simulated a hybrid inorganic/organic interface between MoS_2_ and H_2_Pc with different first-principles approaches, using a combination of *GW*, ΔSCF, and PCM. We found that the energy levels of H_2_Pc are highly sensitive to the screening due to the MoS_2_ layer, substrates, and surrounding molecules, reducing the band gap by up to 2 eV with respect to the isolated molecule. The band gap of MoS_2_, on the other hand, is only reduced by a maximum of 0.5 eV due to substrate polarization. The PBE0 and HSE functionals predict the correct band gap of a MoS_2_ monolayer in the limiting cases of no underlying substrate and of a perfectly screening substrate, respectively, although the absolute energy values are off. These estimates are improved by centering the PBE0 and HSE band gaps around the midgap position predicted by PBE. For an isolated molecule, PBE0 predicts frontier orbital energies that approximate well the corresponding condensed-phase values. These condensed-phase frontier energy levels change only to an insignificant amount when the film is placed on MoS_2_, meaning that the image-charge effect is mainly relevant in the case of low coverage.

Combining these findings, we propose a simple and robust way of determining the energy level alignment at interfaces between the two-dimensional material and a molecular film: centering PBE0/HSE band structures of transition-metal dichalcogenides around the PBE midgap position and relating them to PBE0 results for organic molecules. While we considered only one type of interface, a review of literature reports indicates that this approach is transferable to other iorganic/organic interfaces. Furthermore, we presented an analysis of the wave packet representation of the frontier orbitals of H_2_Pc, which is a tool that allows for an understanding of the electronic structure beyond the energy level alignment. The highest occupied orbital of H_2_Pc can be represented as a superposition of Bloch states with wave vectors around the high-symmetry point K, whereas the lowest unoccupied one is spread uniformly throughout the Brillouin zone. The latter also overlaps unoccupied MoS_2_ states, giving rise to hybrid states that may play a key role in the formation and time evolution of interlayer excitons.

In conclusion, the protocols put forward in this work greatly broaden the range and ease of applicability of density functional theory to hybrid interfaces, avoiding fully atomistic simulations of interfaces and thus the construction of large supercells with exceeding amounts of atoms. At the same time, predictions of energy level alignments are improved and complemented by **k**-resolved insights into hybrid state formation. All required simulations are robust and easy to set up, making the approach well-suited for automatized workflows.

## Supporting Information Available

In the Supporting Information, we expound the theoretical background and report detailed computational parameters, as well as formulas for the *ab initio* calculation of dielectric constants of molecular films and two-dimensional materials. Furthermore, we describe the Δ*W* method of estimating the MoS_2_ band gap renormalization. (PDF)
