# Supplementary material for: Ab Initio Modeling of Mixed-Dimensional Heterostructures: A Path Forward
Source: J Phys Chem Lett. 2024 May 10;15(20):5350–8. doi: 10.1021/acs.jpclett.4c00803 (PMC11129309; doi:10.1021/acs.jpclett.4c00803)
Supplement: Supplementary file 1 — jz4c00803_si_001.pdf [file jz4c00803_si_001.pdf]

SUPPORTING INFORMATION

Ab Initio Modelling of Mixed-Dimensional Heterostructures: A Path Forward

Jannis Krumland*,†,‡ and Caterina Cocchi*,†,‡

†*Institute of Physics, Carl von Ossietzky Universität Oldenburg, 26129 Oldenburg, Germany*

‡*Physics Department and IRIS Adlershof, Humboldt-Universität zu Berlin, 12489 Berlin, Germany*

E-mail: jannis.krumland@physik.hu-berlin.de; caterina.cocchi@uni-oldenburg.de

Theoretical Background and Computational Details

Total-energy calculations for the free-base phthalocyanine (H_2Pc) molecule are performed with density functional theory (DFT),[1,2] employing a modified copy of version 12.2 of Octopus,[3] using the PBE functional[4] and SG15 pseudopotentials.[5] This code represents wave functions and density on a real-space grid, sampling with a spacing of 0.2 Å a simulation box constructed as the union of atom-centered spheres of radius 5 Å. The interface to the polarizable continuum model (PCM) is established by the construction of another sphere-union box, using atom-specific radii (van-der-Waals radii scaled by a factor of 1.05), whose surface is discretized into elements.[6] Each of these elements carries a charge representing part of the polarization charges in the environment. The charge on each element is, in turn, computed from the molecular charges, convolving their electrostatic potential at the boundary elements with the PCM response function.[6] For layered substrates, this response function contains an image term for substrate polarization as implemented in LayerPCM,[7] an extension of PCM for layered environments.

All simulations employing PBE0[8] and HSE[9,10] functionals, as well as all DFT simulations of MoS_2, are conducted with the Quantum ESPRESSO suite,[11] using SG15 (PBE) pseudopotentials.[5] The code expands wave functions $\psi_{\mathbf{K}}$ with crystal momentum $\mathbf{K}$ as

$$\psi_{\mathbf{K}}(\mathbf{r}) \propto e^{i\mathbf{K}\cdot\mathbf{r}} \sum_{\mathbf{G}} \bar{\psi}_{\mathbf{K}}(\mathbf{G}) e^{i\mathbf{G}\cdot\mathbf{r}}, \tag{S1}$$

where $\bar{\psi}_{\mathbf{K}}$ is the plane wave representation of the wave function and the sum runs over the reciprocal lattice vectors $\mathbf{G}$. The summation is cut off at values of $\mathbf{G}$ corresponding to a kinetic energy of 40 Ry. For MoS_2, we use a 24×24 $\mathbf{k}$-grid and, in case of hybrid functionals, an 80 Ry cutoff for the Fock exchange, as well as a 12×12 $\mathbf{q}$-grid. In-plane and out-of-plane lattice constants of 3.18 Å and 15 Å are used, respectively, the former being obtained from geometry optimization, the latter being chosen to include a sufficient amount of vacuum between replicas in the out-of-plane direction.

Eq. (S1) greatly facilitates the calculation of wave packets and unfolded band structures. The molecule is placed in a simulation cell spanned by lattice vectors $\mathbf{A}_i$ that are constructed as superpositions of the primitive lattice vectors $\mathbf{a}_j$ of MoS_2,

$$\begin{pmatrix} \mathbf{A}_1 \\ \mathbf{A}_2 \\ \mathbf{A}_3 \end{pmatrix} = T \begin{pmatrix} \mathbf{a}_1 \\ \mathbf{a}_2 \\ \mathbf{a}_3 \end{pmatrix}, \tag{S2}$$

where T is a 3×3 transformation matrix with integer coefficients. Bloch functions $u\, e^{i\mathbf{K}\cdot\mathbf{r}}$ with $\mathbf{A}_i$-periodic cell functions u are characterized by a Bloch vector $\mathbf{K}$ in the small BZ associated with the $\mathbf{A}_i$ lattice. If this $\mathbf{K}$ is connected to a Bloch vector $\mathbf{k}$ within the large BZ related to the $\mathbf{a}_j$ lattice by a reciprocal lattice vector $\mathbf{G}$ of the $\mathbf{A}_i$ lattice ($\mathbf{k} = \mathbf{K} + \mathbf{G}$), the square modulus of the corresponding wave packet coefficient in Eq. (1) of the main text can be computed as

$$|c_{\mathbf{k}}|^2 = \sum_{\mathbf{g}} |\bar{\psi}_{\mathbf{K}}(\mathbf{G} + \mathbf{g})|^2, \tag{S3}$$

where the sum runs over all reciprocal lattice vectors $\mathbf{g}$ of the $\mathbf{a}_j$ lattice. The unfolded band structure is obtained from the $|c_{n\mathbf{k}}|^2$ of different states $\psi_{n\mathbf{K}}$ as

$$\mathcal{A}(\mathbf{k}, \varepsilon) = \sum_{n} |c_{n\mathbf{k}}|^2 \delta(\varepsilon - \varepsilon_{n\mathbf{K}}), \tag{S4}$$

where $\varepsilon_{n\mathbf{K}}$ is the Kohn-Sham eigenvalue of $\psi_{n\mathbf{K}}$. We perform this calculation in post-processing using an in-house developed code[12] to obtain both the one-dimensional (band structure) and two-dimensional depictions presented in Fig. 3 of the main text. For the two-dimensional representations, the molecule was rotated starting at 15 degrees in steps of 30 degrees, averaging the corresponding spectral functions. For the quasi-degenerate lowest unoccupied orbital, both partner orbitals are averaged over.

To determine the dielectric constants of MoS_2, we employ the random-phase approximation (RPA) on the basis of a PBE electronic structure, obtained with Quantum Espresso and SG15 pseudopotentials with the same **k**-mesh and basis set cutoffs as above. The linear-response calculation is conducted with the Yambo code,[13] including 300 unoccupied bands. The dielectric constants associated with the three-dimensional cell containing the MoS_2 monolayer are transformed into intrinsic material values as described in the following section, yielding $\varepsilon_{\parallel} = 16.52$, $\varepsilon_{\perp} = 10.08$, and a thickness of 5.46 Å. Substrates are modeled as isotropic polarizable media with dielectric constant ε_s, separated from the MoS_2 layer by a vacuum spacer of size 0.69 Å.[14]

The G_0W_0 calculations are performed with Yambo, again using the PBE electronic structure as a starting point, but increasing the out-of-plane lattice constant to 20 Å. A 150-point full-frequency integration is employed to determine the correlation part of the self-energy, using an 80 eV cutoff in the calculation of the RPA-level screening. We repeat the simulation with 100 and 200 unoccupied bands and extrapolate the resulting self-energy correction to infinite bands.[15] We account for the anisotropy in the $\mathbf{q} \to \mathbf{0}$ component of the screening and use a two-dimensional slab Coulomb cutoff.[16]

Visualization of geometries and orbitals was carried out with the XCrySDen program.[17]

Dielectric Models

The dielectric models for the different environments considered in this work as well as the values of the parameters are shown in Fig. 2c). The parameters are determined from first principles as described in the following.

Determination of the Dielectric Constant of a Molecular Film

The dielectric constant ε of a molecular film is determined from DFT in conjunction with the isotropic PCM, using a formula that connects the microscopic polarizability with the

macroscopic dielectric constant. Taking a starting guess for ε, we calculate with OCTOPUS (PBE functional) the dipole moment $\Delta\mathbf{p}(\varepsilon) = (\Delta p_x(\varepsilon), \Delta p_y(\varepsilon), \Delta p_z(\varepsilon))$ induced by a static electric field $E(1,1,1)$ and the corresponding ε-dependent local cavity field,[18] then update ε using

$$\varepsilon = \frac{1}{3}\,\mathrm{Tr}\{\bar{\varepsilon}\} = \frac{4\pi[\Delta p_x(\varepsilon) + \Delta p_y(\varepsilon) + \Delta p_z(\varepsilon)]}{3vE} + 1, \tag{S5}$$

where v is the volume of the PCM cavity, computed with a Monte-Carlo integration. We repeat this procedure until self-consistency, obtaining a value of $\varepsilon = 5.32$ for an H_2Pc film.

Determination of the Dielectric Constants of a Two-Dimensional Material

The dielectric constants $\varepsilon_\parallel$ and $\varepsilon_\perp$ of a polarizable slab replacing a MoS_2 monolayer are determined using the approach outlined in Ref. 19. In brief, we perform linear-response calculations (RPA@PBE) with a simulation cell containing MoS_2 layers separated by a large amount of vacuum. The resulting dielectric constants $\bar{\varepsilon}_\parallel$ and $\bar{\varepsilon}_\perp$ represent an average polarization of the slab and the vacuum spacer. To recover the MoS_2-intrinsic values, we transform $\bar{\varepsilon}_\parallel$ and $\bar{\varepsilon}_\perp$ according to

$$\varepsilon_\parallel = \left(\bar{\varepsilon}_\parallel - 1\right)\frac{c}{d} + 1 \tag{S6}$$

and

$$\varepsilon_\perp = \left[(\bar{\varepsilon}_\perp^{-1} - 1)\frac{c}{d} + 1\right]^{-1}, \tag{S7}$$

which can be derived from electrostatic boundary conditions.[20] In these equations, c is the height of the simulation cell and d is the thickness of the slab, which is adjusted until the

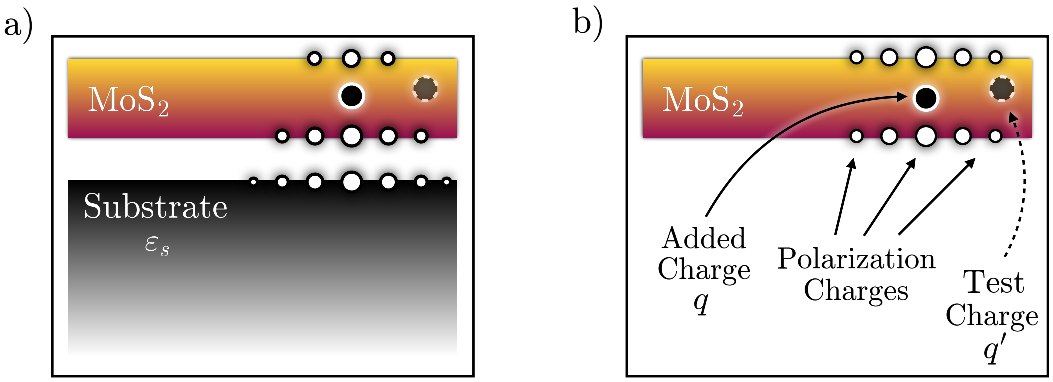

Figure S1: Polarization charges emerging as a consequence of a charge q added in the MoS_2 layer

long-range electrostatic image potential of the slab,[7]

$$v_{\text{img}}(z) = -\frac{1}{2}\left(\varepsilon_\perp \varepsilon_\parallel - 1\right) \int_0^\infty \mathrm{d}q_\parallel \, \frac{e^{-2q_\parallel (z-z_0)}}{1 + \varepsilon_\perp \varepsilon_\parallel + 2(\varepsilon_\perp \varepsilon_\parallel)^{1/2} \coth\!\big(q_\parallel (\varepsilon_\parallel/\varepsilon_\perp)^{1/2} d\big)}, \tag{S8}$$

smoothly transitions into the plane-averaged PBE exchange-correlation potential. This approach corresponds to the explicit construction of a smooth and consistent exchange-correlation self-energy.[21,22] We end up with the values $\varepsilon_\parallel = 16.52$, $\varepsilon_\perp = 10.08$, and $d = 5.46$ Å.

Bandgap Renormalization For MoS_2: The ΔW model

The renormalization of the band gap of MoS_2 is estimated with an electrostatic model. We consider the setup in Fig. S1a), where a charge q is added at the position $\mathbf{r}$ within the substrate-sustained MoS_2 monolayer, parametrized as specified in Fig. 2c) in the main text. As shown in Ref. 14, the corresponding Poisson's equation can be solved without further approximations to determine the full electrostatic potential φ, including polarization contributions. The potential $\varphi(\mathbf{r}')$ felt by a test charge q' at $\mathbf{r}'$ is proportional to the statically screened interaction W between q and q', $W(\mathbf{r}, \mathbf{r}') = \varphi(\mathbf{r}')/q$. Combining this result with the screened interaction W_0 for a reference setup without substrate [Fig. S1b)], we obtain a self-energy correction to the GW band structure of an isolated monolayer due to substrate

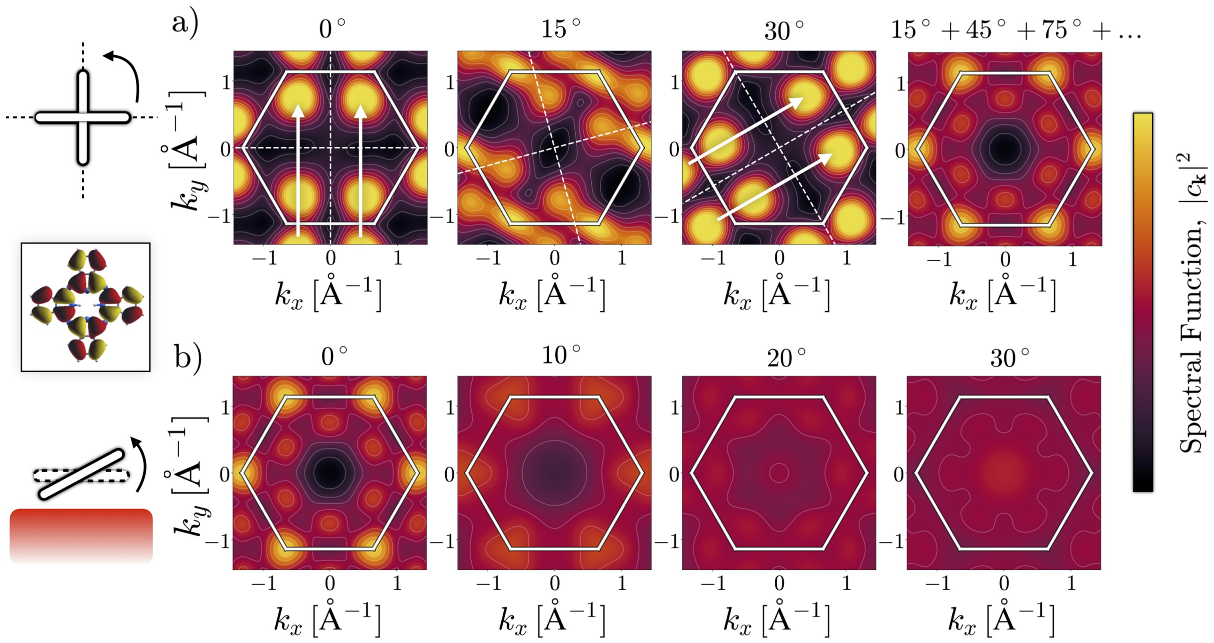

Figure S2: a) Wave packet representation of the highest occupied orbital of H_2Pc in the MoS_2 Brillouin zone upon rotation around the principal axis. The arrows indicate the folding processes that map features back into the first BZ, based on comparison to the unfolded momentum map in Fig. 8 of Ref. 34. b) Evolution of the azimuthally averaged wave packet upon out-of-plane rotation.

polarization as[23]

$$\Delta\Sigma = \pm\frac{1}{2}\lim_{\mathbf{r}'\to\mathbf{r}}[W(\mathbf{r},\mathbf{r}') - W_0(\mathbf{r},\mathbf{r}')] \tag{S9}$$

where "+" and "-" refer to occupied and unoccupied bands, respectively, and we set $\mathbf{r}$ to the center of the monolayer. W_0 has to be subtracted as a corresponding contribution is already accounted for in the GW calculation of the isolated monolayer.

The experimental ionization potential of substrate-sustained monolayer MoS_2 quoted in the main text (6.10 eV) is the average of the following results from photoelectron spectroscopy: 5.90 eV,[24] 5.95 eV,[25] 6.10 eV,[26] 6.28 eV,[27] 6.38 eV,[28] 6.30 eV,[28] 6.23 eV,[28] 6.26 eV,[29] 6.20 eV,[29] 5.77 eV,[30] 5.70 eV,[31] 6.18 eV,[32] and 6.10 eV[33] (standard deviation = 0.20 eV).

References

(1) Hohenberg, P.; Kohn, W. Inhomogeneous Electron Gas. *Phys. Rev.* **1964**, *136*, B864–B871.

(2) Kohn, W.; Sham, L. J. Self-Consistent Equations Including Exchange and Correlation Effects. *Phys. Rev.* **1965**, *140*, A1133–A1138.

(3) Tancogne-Dejean, N. et al. Octopus, a computational framework for exploring light-driven phenomena and quantum dynamics in extended and finite systems. *J. Chem. Phys.* **2020**, *152*, 124119.

(4) Perdew, J. P.; Burke, K.; Ernzerhof, M. Generalized Gradient Approximation Made Simple. *Phys. Rev. Lett.* **1996**, *77*, 3865–3868.

(5) Schlipf, M.; Gygi, F. Optimization algorithm for the generation of ONCV pseudopotentials. *Comput. Phys. Commun.* **2015**, *196*, 36–44.

(6) Tomasi, J.; Mennucci, B.; Cammi, R. Quantum Mechanical Continuum Solvation Models. *Chem. Rev.* **2005**, *105*, 2999–3094.

(7) Krumland, J.; Gil, G.; Corni, S.; Cocchi, C. LayerPCM: An implicit scheme for dielectric screening from layered substrates. *J. Chem. Phys.* **2021**, *154*, 224114.

(8) Adamo, C.; Barone, V. Toward reliable density functional methods without adjustable parameters: The PBE0 model. *J. Chem. Phys.* **1999**, *110*, 6158–6170.

(9) Heyd, J.; Scuseria, G. E.; Ernzerhof, M. Hybrid functionals based on a screened Coulomb potential. *J. Chem. Phys.* **2003**, *118*, 8207–8215.

(10) Heyd, J.; Scuseria, G. E.; Ernzerhof, M. Erratum: "Hybrid functionals based on a screened Coulomb potential" [J. Chem. Phys. 118, 8207 (2003)]. *J. Chem. Phys.* **2006**, *124*, 219906.

(11) Giannozzi, P. et al. Advanced capabilities for materials modelling with Quantum ESPRESSO. *J. Phys.: Condens. Matter.* **2017**, *29*, 465901.

(12) Krumland, J.; Cocchi, C. Conditions for electronic hybridization between transition-metal dichalcogenide monolayers and physisorbed carbon-conjugated molecules. *Electronic Structure* **2021**, *3*, 044003.

(13) Sangalli, D.; Ferretti, A.; Miranda, H.; Attaccalite, C.; Marri, I.; Cannuccia, E.; Melo, P.; Marsili, M.; Paleari, F.; Marrazzo, A.; et al., Many-body perturbation theory calculations using the yambo code. *J. Phys.: Condens. Matter.* **2019**, *31*, 325902.

(14) Krumland, J.; Cocchi, C. Electronic Structure of Low-Dimensional Inorganic/Organic Interfaces: Hybrid Density Functional Theory, G 0 W 0, and Electrostatic Models. *Phys. Status Solidi A n/a*, 2300089.

(15) Klimeš, J. c. v.; Kaltak, M.; Kresse, G. Predictive *GW* calculations using plane waves and pseudopotentials. *Phys. Rev. B* **2014**, *90*, 075125.

(16) Rozzi, C. A.; Varsano, D.; Marini, A.; Gross, E. K. U.; Rubio, A. Exact Coulomb cutoff technique for supercell calculations. *Phys. Rev. B* **2006**, *73*, 205119.

(17) Kokalj, A. XCrySDen - A New Program for Displaying Crystalline Structures and Electron Densities. *J. Mol. Graphics Modell.* **1999**, *17*, 176–179.

(18) Gil, G.; Pipolo, S.; Delgado, A.; Rozzi, C. A.; Corni, S. Nonequilibrium Solvent Polarization Effects in Real-Time Electronic Dynamics of Solute Molecules Subject to Time-Dependent Electric Fields: A New Feature of the Polarizable Continuum Model. *J. Chem. Theory. Comput.* **2019**, *15*, 2306–2319, PMID: 30860829.

(19) Cocchi, C.; Guerrini, M.; Krumland, J.; Nguyen, N. T.; Valencia, A. M. Modeling the electronic structure of organic materials: a solid-state physicist's perspective. *J. Phys.Materials* **2022**, *6*, 012001.

(20) Aspnes, D. E. Local-field effects and effective-medium theory: A microscopic perspective. *Am. J. Phys.* **1982**, *50*, 704–709.

(21) Lang, N. D.; Kohn, W. Theory of Metal Surfaces: Induced Surface Charge and Image Potential. *Phys. Rev. B* **1973**, *7*, 3541–3550.

(22) Eguiluz, A. G.; Hanke, W. Evaluation of the exchange-correlation potential at a metal surface from many-body perturbation theory. *Phys. Rev. B* **1989**, *39*, 10433–10436.

(23) Cho, Y.; Berkelbach, T. C. Environmentally sensitive theory of electronic and optical transitions in atomically thin semiconductors. *Phys. Rev. B* **2018**, *97*, 041409.

(24) Tsai, M.-L.; Su, S.-H.; Chang, J.-K.; Tsai, D.-S.; Chen, C.-H.; Wu, C.-I.; Li, L.-J.; Chen, L.-J.; He, J.-H. Monolayer MoS2 Heterojunction Solar Cells. *ACS Nano* **2014**, *8*, 8317–8322, PMID: 25046764.

(25) Chang, Y.-H. et al. Monolayer MoSe2 Grown by Chemical Vapor Deposition for Fast Photodetection. *ACS Nano* **2014**, *8*, 8582–8590, PMID: 25094022.

(26) Obaidulla, S. M.; Habib, M. R.; Khan, Y.; Kong, Y.; Liang, T.; Xu, M. MoS2 and Perylene Derivative Based Type-II Heterostructure: Bandgap Engineering and Giant Photoluminescence Enhancement. *Advanced Materials Interfaces* **2020**, *7*, 1901197.

(27) Park, S.; Mutz, N.; Kovalenko, S. A.; Schultz, T.; Shin, D.; Aljarb, A.; Li, L.-J.; Tung, V.; Amsalem, P.; List-Kratochvil, E. J. W.; Stähler, J.; Xu, X.; Blumstengel, S.; Koch, N. Type-I Energy Level Alignment at the PTCDA—Monolayer MoS2 Interface Promotes Resonance Energy Transfer and Luminescence Enhancement. *Advanced Science* **2021**, *8*, 2100215.

(28) Park, S.; Schultz, T.; Xu, X.; Wegner, B.; Aljarb, A.; Han, A.; Li, L.-J.; Tung, V. C.; Amsalem, P.; Koch, N. Demonstration of the key substrate-dependent charge transfer

mechanisms between monolayer MoS2 and molecular dopants. *Communications Physics* **2019**, *2*, 109.

(29) Park, S.; Mutz, N.; Schultz, T.; Blumstengel, S.; Han, A.; Aljarb, A.; Li, L.-J.; List-Kratochvil, E. J. W.; Amsalem, P.; Koch, N. Direct determination of monolayer MoS2 and WSe2 exciton binding energies on insulating and metallic substrates. *2D Materials* **2018**, *5*, 025003.

(30) Keyshar, K.; Berg, M.; Zhang, X.; Vajtai, R.; Gupta, G.; Chan, C. K.; Beechem, T. E.; Ajayan, P. M.; Mohite, A. D.; Ohta, T. Experimental Determination of the Ionization Energies of MoSe2, WS2, and MoS2 on SiO2 Using Photoemission Electron Microscopy. *ACS Nano* **2017**, *11*, 8223–8230, PMID: 28723073.

(31) Mutz, N.; Park, S.; Schultz, T.; Sadofev, S.; Dalgleish, S.; Reissig, L.; Koch, N.; List-Kratochvil, E. J. W.; Blumstengel, S. Excited-State Charge Transfer Enabling MoS2/Phthalocyanine Photodetectors with Extended Spectral Sensitivity. *J. Phys. Chem. C* **2020**, *124*, 2837–2843.

(32) Raoufi, M.; Chandrabose, S.; Wang, R.; Sun, B.; Zorn Morales, N.; Shoaee, S.; Blumstengel, S.; Koch, N.; List-Kratochvil, E.; Neher, D. Influence of the Energy Level Alignment on Charge Transfer and Recombination at the Monolayer-MoS2/Organic Hybrid Interface. *J. Phys. Chem. C* **2023**, *127*, 5866–5875.

(33) Kong, Y.; Obaidulla, S. M.; Habib, M. R.; Wang, Z.; Wang, R.; Khan, Y.; Zhu, H.; Xu, M.; Yang, D. Interlayer exciton emission in a MoS2/VOPc inorganic/organic van der Waals heterostructure. *Mater. Horiz.* **2022**, *9*, 1253–1263.

(34) Schönauer, K.; Weiss, S.; Feyer, V.; Lüftner, D.; Stadtmüller, B.; Schwarz, D.; Sueyoshi, T.; Kumpf, C.; Puschnig, P.; Ramsey, M. G.; Tautz, F. S.; Soubatch, S. Charge transfer and symmetry reduction at the CuPc/Ag(110) interface studied by photoemission tomography. *Phys. Rev. B* **2016**, *94*, 205144.
